# Comparative Transcriptome Analysis Suggests Key Roles for 5-Hydroxytryptamlne Receptors in Control of Goose Egg Production

**DOI:** 10.3390/genes11040455

**Published:** 2020-04-22

**Authors:** Qingyuan Ouyang, Shenqiang Hu, Guosong Wang, Jiwei Hu, Jiaman Zhang, Liang Li, Bo Hu, Hua He, Hehe Liu, Lu Xia, Jiwen Wang

**Affiliations:** Farm Animal Genetic Resources Exploration and Innovation Key Laboratory of Sichuan Province, Sichuan Agricultural University, Chengdu 611130, China; oyqy222@163.com (Q.O.); shenqianghu@gmail.com (S.H.); guosong.wang@tamu.edu (G.W.); hujiwei1990@126.com (J.H.); zjm960521@163.com (J.Z.); Liliang@sicau.edu.cn (L.L.); hubolirong@163.com (B.H.); hehua023@126.com (H.H.); Liuee1985@sicau.edu.cn (H.L.); xlaza@sicau.edu.cn (L.X.)

**Keywords:** goose, lncRNA, mRNA, ovary, transcriptome sequencing

## Abstract

To date, research on poultry egg production performance has only been conducted within inter or intra-breed groups, while those combining both inter- and intra-breed groups are lacking. Egg production performance is known to differ markedly between Sichuan white goose (*Anser cygnoides*) and Landes goose (*Anser anser*). In order to understand the mechanism of egg production performance in geese, we undertook this study. Here, 18 ovarian stromal samples from both Sichuan white goose and Landes goose at the age of 145 days (3 individuals before egg production initiation for each breed) and 730 days (3 high- and low egg production individuals during non-laying periods for each breed) were collected to reveal the genome-wide expression profiles of ovarian mRNAs and lncRNAs between these two geese breeds at different physiological stages. Briefly, 58, 347, 797, 777, and 881 differentially expressed genes (DEGs) and 56, 24, 154, 105, and 224 differentially expressed long non-coding RNAs (DElncRNAs) were found in LLD vs. HLD (low egg production Landes goose vs. high egg production Landes goose), LSC vs. HSC (low egg production Sichuan White goose vs. high egg production Sichuan white goose), YLD vs. YSC (young Landes goose vs. young Sichuan white goose), HLD vs. HSC (high egg production Landes goose vs. high egg production Sichuan white goose), and LLD vs. LSC (low egg production Landes goose vs. low egg production Sichuan white goose) groups, respectively. Functional enrichment analysis of these DEGs and DElncRNAs suggest that the “neuroactive ligand–receptor interaction pathway” is crucial for egg production, and particularly, members of the 5-hydroxytryptamine receptor (HTR) family affect egg production by regulating ovarian metabolic function. Furthermore, the big differences in the secondary structures among HTR1F and HTR1B, HTR2B, and HTR7 may lead to their different expression patterns in goose ovaries of both inter- and intra-breed groups. These results provide novel insights into the mechanisms regulating poultry egg production performance.

## 1. Introduction

Compared to chickens and ducks, geese have poorer egg production performance, which severely impedes the development of the goose industry [1]. In goose, there are also inter-breed and intra-breed differences in egg production performance. For instance, compared with the annual egg production of about 80 in Sichuan white geese (SWG), the annual egg production of Landes goose (LG) is 40~60 [2,3]. To our knowledge, most studies on poultry egg production performance have focused on the mechanisms underlying either inter-breed or intra-breed differences [4,5,6]. However, there lack studies that include comparisons not only between different breeds differing in egg numbers but also between different ages within the same breed to better reveal the mechanisms controlling egg production performance. Also, 145 days is the time before the goose begins to lay eggs, and 730 days old represents two years of egg-laying. The combined analysis of the two ages can help us to systematically understand the goose’s egg production performance.

The hypothalamic–pituitary–gonadal (HPG) axis initiates the maturation of the ovary, which is the organ responsible for laying eggs in poultry. So far, the emerging -omic studies mainly adopted either the intact ovary or its components such as mature follicles to explore the molecular mechanisms controlling poultry egg production performance. For example, transcriptome sequencing of the ovaries from hens with different egg numbers revealed an important role of the neuroactive ligand–receptor interaction pathway in the regulation of egg production performance [7]. Meanwhile, a key role for lipid metabolism in the process of follicle selection has also been recently identified by transcriptome sequencing of goose follicles at different developmental stages [8]. After hatching, the ovary of poultry differentiates into two parts, including the external cortex and internal medulla [9], with the cortex being composed of the stroma and numerous follicles [10]. However, although the ovarian stroma is essential for ovarian follicle maturation and consequently affects egg numbers, little sequencing has been carried out in order to further reveal the mechanisms controlling egg production performance.

With the rapid development of high-throughput sequencing technologies, long non-coding RNAs (lncRNAs) have gradually attracted more attention. LncRNA is a kind of non-coding RNA whose transcript length exceeds 200 nt and is known to regulate reproductive performance by participating in gametogenesis [11,12], hormone regulation, sex determination [13], and gonadal development [14]. Nevertheless, combined analysis of both mRNA and lncRNA expression profiling has not been reported in the ovarian stroma of poultry. Therefore, the present study aims to investigate the mRNA and lncRNA transcriptome profiles in the ovarian stroma of LG and SWG at 145 and 730 days of age. Comparative analysis of the inter-breed and intra-breed differences in the mRNA and lncRNA transcriptomes is expected to help elucidate the mechanisms regulating poultry egg production performance.

## 2. Materials and Methods

### 2.1. Ethics Statement

All experimental procedures that involved animal manipulation were approved by the Faculty Animal Care and Use Committee of Sichuan Agricultural University (Ya’an, Sichuan, China) under permit no. DKY20170913. 

### 2.2. Experimental Design and Sample Collection

The SWG and LG were obtained from the Waterfowl Breeding Experimental Farm of Sichuan Agricultural University. Three of SWGs and LGs, aged 145 days, were selected for sampling. For individuals from the same breed, they had similar body weights (3.13–3.77 kg) and were in a similar physiological state approximately 2–3 months before egg-laying. At the age of 730 days, according to the egg production record, the geese from the highest 30% egg production were divided into the high egg production performance group, and the geese from the lowest 30% production were divided into the low egg production performance group. In each group, three geese, with similar body weight (3.31–3.77 kg), physiology (main feathers are fully crossed), and starting laying time, were selected to be in the production cessation period. After around 12-h fasting, all selected geese were slaughtered by cervical dislocation, and the intact ovaries were removed immediately after slaughter. After the removal of visible follicles residing on the ovarian surface, the ovarian stroma was cut into pieces and stored at −80 °C after snap-freezing in liquid nitrogen until analysis.

### 2.3. RNA Isolation and Sequencing

Following the manufacture’s instruction, total RNA was extracted from the ovarian stroma of each individual using the RNeasy Mini Kit (Qiagen, Beijing, China). The RNA integrity was determined by an Agilent Bioanalyzer 2100 (Agilent Technologies, Santa Clara, CA, USA). The RNAs with an average RIN value of 8.96 (7.8~10.0) were sent to generate libraries by Novogene (Novogene, Tianjin, China). All libraries were sequenced by the Novogene Illumina PE 150. RNA- and lncRNA-seq data used in the current study are available at the BioProject 598883 in NCBI (National Center for Biotechnology Information). The clean reads were obtained after the filtration of low-quality reads using standard quality control by FastaQC software.

### 2.4. Transcriptome Alignment and Assembly

Clean reads were mapped to the goose reference genome (anscyg_prjna183603_v1.0) using the HISAT2 (version 2.1.0) software [15]. The output SAM (sequencing alignment/mapping) file was converted to a BAM (binary alignment/mapping) file and sorted using SAMtools (0.1.19-44428cd) [16]. StringTie (version 1.3.3b) [17] was used to assemble transcripts after integrating all individual transcripts and quantitatively expressed genes. Gffcompare (version 0.10.1) [18] was used to examine the assembly of the transcripts associated with annotated genomes.

### 2.5. Identification of the lncRNAs

We determined the fragment per kilobase of exon per million fragments mapped (FPKM) value of each transcript by StringTie and retained the transcripts with FPKM > 0.5 from each sample. Then, the remaining transcripts were merged. Then, the transcripts with a length of >200 nt, exons >2, coverage >3, and class_code = “j” (potentially novel isoform: At least one splice junction is shared with a reference transcript), “i” (a transcript falling entirely within a reference intron, potential intronic lncRNA), “u” (a transcript from an intergenic region, potential intergenic lncRNA), “x” (a transcript overlapping with the opposite strand of reference transcript, potential antisense lncRNA), or “o” (generic exonic overlap with a reference transcript) were retained. CPC2 [19] and CNCI [20] were used to exclude coding genes, and the rest were considered as lncRNAs.

### 2.6. Identification of DEGs and DE lncRNAs

Eighteen samples were divided into five groups based on either breed or egg production performance, and the grouping information is listed in Table 1. Gene expression levels were quantified using Ballgown [21]. The DEGs and DE lncRNAs were filtered based on *p*-values <0.05 and |log2FC| > 1.

### 2.7. Analysis of the Identified DEGs and DE lncRNAs

The coding genes located at around 100 kb upstream of each DE lncRNA were considered potential cis-regulated target genes. Correlation analysis between expression of all coding genes and lncRNAs was performed using the R package Hmisc. LncRNA–mRNA pairs with correlation coefficients >0.80 and *p* < 0.05 were identified as correlated pairs. Gene ontology enrichment analysis software tools (GOEAST) [22] was used to analyze the Gene Ontology (GO) functions. KOBAS3.0 [23] was used to analyze the Kyoto Encyclopedia of Genes and Genomes (KEGG) functions.

### 2.8. RT-qPCR Validation of the DE lncRNAs and Their Target Genes

The DEGs and DE lncRNAs were randomly selected from the results and further verified by RT-PCR. Previously, total RNA extracted from the stroma of the ovary was reverse transcribed into cDNA using a RevertAid First Strand cDNA Synthesis Kit kit (Thermo, MA, USA). Primer 5.0 was used to design the primers (Table 2). A BLAST search against the reference genome was then carried out to confirm primers were specific for the intended target genes. SYBR Green PCR SuperMix (Bio-Rad, Hercules, CA, USA) and a Bio-Rad CFX96 real-time PCR detection system (Bio-Rad, Hercules, CA, USA) were used for RT-PCR, and each sample was assayed three times. *β*-actin [24] was used as a housekeeping gene. The 2^−ΔΔCT^ method was used for normalization of the qPCR results, after which the normalized data was used for statistical analysis, and *p* < 0.05 was considered significantly different.

### 2.9. Sequence Analysis of the HTR Family Genes

DNAMAN software was used to compare the coding sequence (CDS) of the *HTR1B*, *HTR1F*, *HTR2B*, and *HTR7* genes. After the nucleic acid sequence was translated into protein, the ExPASy Proteomics Server [25] was used to predict the physicochemical properties of the protein, and then, the transmembrane capacity was predicted. Functional domains of the HTR1B, HTR1F, HTR2B, and HTR7 proteins were predicted online using CDD-Search in NCBI. The secondary and tertiary structures of the proteins were predicted by SOPMA online software [26] and SWISS-MODEL [27], respectively.

## 3. Results

### 3.1. Characteristics of All Obtained Ovarian Transcriptomes

**Figure 1:**
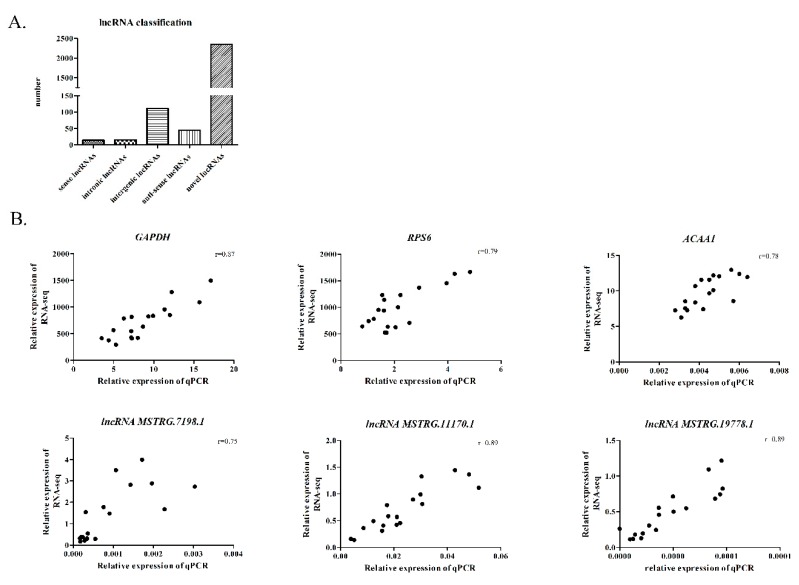
Profile of the RNA sequencing. (**A**) Classification of the long non-coding RNAs (lncRNAs) in our study. (**B**) Validation of three differentially expressed genes (DEGs) and three differentially expressed lncRNAs (DElncRNAs) by qPCR.

A total of 952,812,144 raw reads were obtained from 18 samples through sequencing. Each sample yielded 52,113,693 clean reads after strict filtering. The Q20 (percentage of reads with a Phred quality value >20) and Q30 (percentage of reads with a Phred quality value >30) of the clean reads ranged from 95.09~97.34% and 88.50~93.33%, respectively. The mapping rate of the 18 samples ranged from 74.43~83.16% (Table S1). After re-assembly of the transcripts, a total of 25,883 genes and 2538 lncRNAs were identified from the 18 samples for subsequent analysis after screening. These lncRNAs included 14 sense lncRNAs (from class_code “o” transcripts), 15 intronic lncRNAs (from class_code “i” transcripts), 111 intergenic lncRNAs (from class_code “u” transcripts), 45 antisense lncRNAs (from class_code “x” transcripts) and 2353 novel lncRNAs (from class_code “j” transcripts) (Figure 1A). The average exon number and length of the lncRNAs were 11.5 and 4978 bp, respectively. In addition, three protein-coding genes and three lncRNAs were randomly selected for RT-PCR analysis to confirm our RNA-seq accuracy. A high correlation of the log2FC (r > 0.75) between RNA-seq and RT-PCR was observed (Figure 1B).

### 3.2. Identification of DEGs and DE lncRNAs Between Either Different Breeds or Different Egg Production Performance within the Same Breed

**Figure 2:**
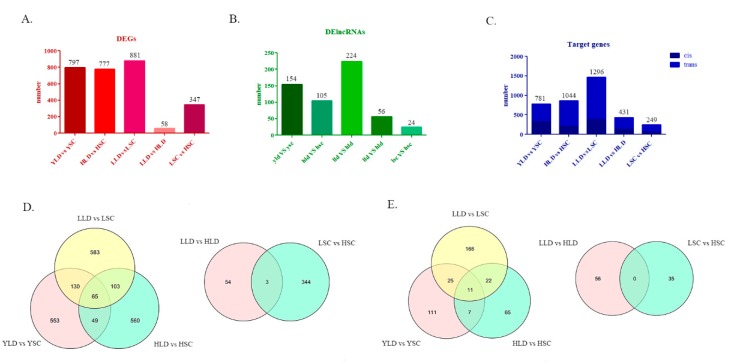
Histogram of the number of DEGs (**A**), DE lncRNAs (**B**), and target genes (**C**) in the different groups. Venn diagram of the DEGs (**D**) and the DE lncRNAs (**E**) in the different groups.

Fifty-eight and 347 DEGs were identified in the comparisons of LLD vs. HLD and LSC vs. HSC, respectively (Figure 2A; Table S2), and 3 overlapping DEGs were found between these two comparisons (Figure 2D). However, there were 797,777 and 881 DEGs present between YLD vs. YSC, HLD vs. HSC, and LLD vs. LSC, respectively (Figure 2A; Table S2), and 65 overlapping DEGs were found between them (Figure 2D). The number of DE lncRNAs between the breeds was much higher than that between individuals at the same age differing in egg production performance within the same breed. There were 154,105, and 224 DE lncRNAs present between YLD vs. YSC, HLD vs. HSC, and LLD vs. LSC, respectively (Figure 2B; Table S3). However, there were only 56 and 24 DE lncRNAs present in LLD vs. HLD and LSC vs. HSC, respectively (Figure 2B; Table S3). Eleven DE lncRNAs overlapped in the three groups between breeds, while there were no overlapping DE lncRNAs in LLD vs. HLD and LSC vs. HSC groups (Figure 2E). The number of cis-regulated target genes between YLD vs. YSC, HLD vs. HSC, LLD vs. LSC, LLD vs. HLD, and LSC vs. HSC was 335,380, 203,133, and 63, respectively (Figure 2C; Table S5). Furthermore, 446,664, 1093, 298, and 186 trans-regulated genes between YLD vs. YSC, HLD vs. HSC, LLD vs. LSC, LLD vs. HLD, and LSC vs. HSC were predicted, respectively (Figure 2C; Table S6).

### 3.3. Functional Analysis of the Identified DEGs Reveals Key Roles for HTR in Control of Egg Production Performance

**Figure 3:**
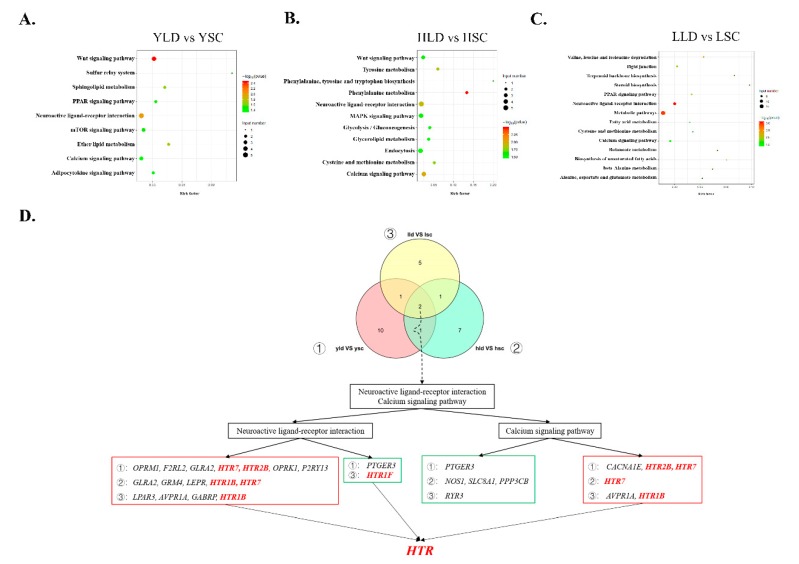
Kyoto Encyclopedia of Genes and Genomes (KEGG) pathways enriched in the DEGs found between the breeds. The KEGG pathways of the DEGs between YLD vs. YSC (**A**), HLD vs. HSC (**B**), and LLD vs. LSC (**C**). (**D**) The neuroactive ligand–receptor interaction pathway and calcium signaling pathway overlapped in the three groups. The red box represents significantly upregulated DEGs, and the green box represents significantly downregulated DEGs. All the DEGs associated with HTR are shown in red.

We performed functional enrichment analysis of each set of DEGs. In the YLD vs. YSC group, most significantly enriched pathways were related to metabolism, including steroid metabolism (steroid biosynthesis pathway), fatty acid metabolism (PPAR signaling pathway, biosynthesis of unsaturated fatty acids and fatty acid metabolism), and amino acid metabolism (valine, leucine, and isoleucine degradation/β-alanine metabolism/alanine, aspartate and glutamate metabolism/cysteine and methionine metabolism) (Figure 3A). Most of these DEGs related to metabolism were significantly upregulated in LG (Table S2). The Wnt signaling pathway was enriched in HLD vs. HSC and LLD vs. LSC groups, and these DEGs were significantly upregulated in the SWG group (Table S2). Other KEGG pathways were mostly related to cell cycle and glycol metabolism (Figure 3B,C). Notably, the calcium signaling and neuroactive ligand–receptor interaction pathways were significantly enriched in YLD vs. YSC, HLD vs. HSC, and LLD vs. LSC groups. Except for *PTGER3* and *HTR1F*, all DEGs enriched in the neuroactive ligand–receptor interaction pathway were significantly upregulated in the SWG group (Figure 3D). The expression of *HTR7* and *GLRA2* at 145 and 730 days were both significantly different. *HTR2B* was differentially expressed between the two breeds only at 145 days of age. *HTR1B* and *HTR1F* was differentially expressed between the two breeds only at 730 days of age.

### 3.4. The DE lncRNAs Target Members of the HTR Family to Regulate Inter-Breed Differences in Egg Production Performance

**Figure 4:**
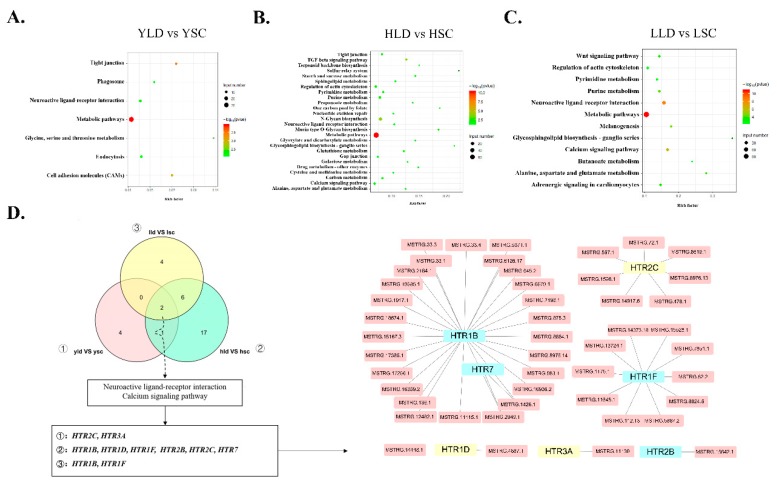
KEGG pathways enriched in the DE lncRNAs between the breeds. The KEGG pathways enriched in the DE lncRNAs between YLD vs. YSC (**A**), HLD vs. HSC (**B**), and LLD vs. LSC (**C**). (**D**) The neuroactive ligand–receptor interaction pathway and calcium signaling pathway overlapped in the three groups. Target genes belonging to the *HTR* family in each group are listed separately. *HTR1B*, *HTR2B*, *HTR1F*, and *HTR7* were differentially expressed in at least one group (blue module), and *HTR2C*, *HTR1D*, and *HTR3A* were not differentially expressed in each group (yellow module). The DE lncRNAs are represented by pink ellipses.

Similarly to protein-coding genes, the predicted target genes of DE lncRNAs identified between different breeds were also predicted to be involved in calcium signaling and neuroactive ligand–receptor interaction pathways at the two ages (Figure 4D). Moreover, metabolism-related pathways were enriched at the two ages. Furthermore, six of the same KEGG pathways in the two groups at 730 days were related to metabolism, in addition to the calcium signaling pathway and regulation of the actin cytoskeleton. Subsequently, we constructed an interaction network for *HTR* family target genes and their corresponding DE lncRNAs. *HTR3A* was cis-regulated by lncRNA MESTG.11130, and other effect pairs were predicted to show trans-regulation. Notably, lncRNA.MSTRG.1426.1 targets both *HTR7* and *HTR1B*. *HTR2B* was only regulated by lncRNA.MSTRG.15642.1. *HTR1F* is co-regulated by multiple lncRNAs. The DE lncRNAs also target *HTR2C*, *HTR1D*, and *HTR3A*. However, the FPKMs of *HTR2C*, *HTR1D*, and *HTR3A* in each group were not significantly different.

### 3.5. HTR1B Regulates Intra-Breed Difference in Egg Production Performance within SWG as Opposed to LG

**Figure 5:**
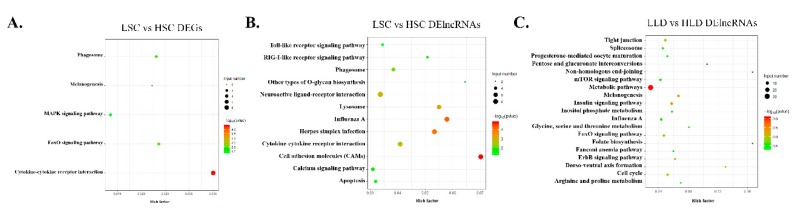
Functional analysis of the DEGs and DE lncRNAs in intra-breed groups with different egg production performances. KEGG pathways significantly enriched by the DEGs (**A**) and the DE lncRNAs (**B**) in the LSC vs. HSC group and the DE lncRNAs (**C**) in the LLD vs. HLD group.

There were few DEGs identified between individuals at the same age differing in egg production performance in LG. By comparison, there were more DE lncRNAs identified between individuals at the same age differing in egg production performances in LG, and these DEGs were significantly enriched in the insulin, metabolic, and melanogenesis pathways, as suggested by functional enrichment analysis (Figure 5C). Compared to LG, much more DEGs but fewer DE lncRNAs were identified in individuals of the same age differing in egg production performance in SWG. The DEGs in LLD vs. HLD and LSC vs. HSC groups had some hierarchical relationships in biological process through GO analysis (Figure S1). The DEGs and DE lncRNAs of the SWG group were enriched in both the cytokine–cytokine receptor interaction and phagosome pathways (Figure 5A,B). The influenza A pathway was enriched by the DE lncRNAs of the LSC vs. HSC and LLD vs. HLD groups (Figure 5B,C). However, neuroactive ligand–receptor interaction and the calcium signaling pathway were only enriched in LSC vs. HSC group, as shown by the functional analysis of the DE lncRNAs (Figure 5B). In particular, *HTR1B* was regulated by lncRNA MSTRG.136.1, which is a difference related to the different egg production performances in SWG.

### 3.6. Structure Prediction and Expression Validation of Several Members of the HTR Gene Family

**Figure 6:**
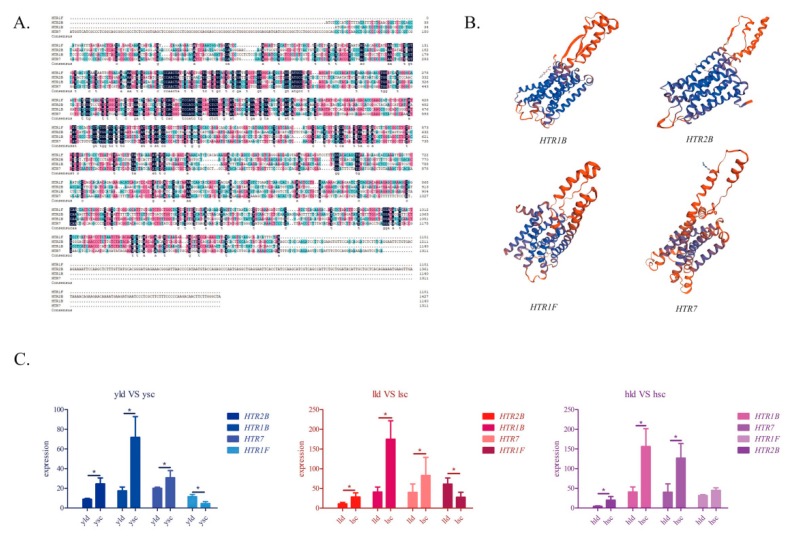
Sequence analysis and expression verification of *HTR1B, HTR1F, HTR2B*, and *HTR7*. (**A**) CDS region sequence alignment; 100% consistent sequences are shown in dark blue, 75% consistent sequences are shown in pink, and 50% consistent sequences are shown in light blue; (**B**) the three-dimensional protein structures of HTR1B, HTR2B, HTR1F, and HTR7; (**C**) gene expression comparison in the two breeds of goose, * represents a significant difference between the two groups of data (*p* < 0.05).

Although *HTR1B*, *HTR2B*, *HTR1F*, and *HTR7* showed differential expression in the two breeds, their expression patterns were not identical. Furthermore, we used bioinformatics to analyze their sequences. All of these genes contain a tm_GPCRs superfamily domain. The CDS regions of *HTR2B* are 50.05%, 49.95%, and 51.08% similar to those of *HTR7*, *HTR1F*, and *HTR1B*, respectively. Additionally, *HTR7* was 53.88% and 57.46%, similar to *HTR1F* and *HTR1B*, respectively. *HTR1F* and *HTR1B* had the highest similarity (60.17%). The proteins translated by the four genes were predicted to be hydrophobic proteins, and all of them were stable proteins except HTR7 (instability index = 45.26). We predicted their secondary structure and found that the HTR1F protein had the highest proportion of α helices and the lowest percentage of β turns and random coils. Three-dimensional structure models of the four proteins were constructed based on homology (Figure 6B). In a specific region of each gene, primers were designed to detect the expression of the four genes. *HTR1B*, *HTR2B*, and *HTR7* were significantly upregulated in the ovarian stroma of SWG, as shown by RT-PCR, at the two time points. In contrast, *HTR1F* was significantly downregulated in the ovarian stroma of SWG between YLD vs. YSC and LLD vs. LSC, but the difference was not significant in the HLD vs. HSC group.

## 4. Discussion

The annual egg production rate of SWG was significantly higher than that of LG. Consistent with this observation, many DEGs and DE lncRNAs were identified in the ovarian stroma between SWG and LG. Subsequent analysis suggested that the neuroactive ligand–receptor interaction pathway was significantly enriched by both the DEGs and DE lncRNAs between SWG and LG at two ages. Transcriptome studies in poultry [7], pig [28], goat [29], and zebrafish [30] have also demonstrated the important role of this pathway in the control of reproductive activities. Furthermore, almost all DEGs enriched in this pathway, including *OPRM1*, *F2RL2*, *GLRA2*, *HTR7*, *HTR2B*, *OPRK1*, *P2RY13*, *GRM4*, *LEPR*, *HTR1B*, *LPAR3*, *AVPR1A*, and *GABRP*, were significantly upregulated in the ovarian stroma of SWG. As a receptor for oxytocin, *AVPR1A* was shown to mediate the secretion of ACTH and PRL by binding AVT to finely regulate ovulation in chickens [31]. Expression levels of *OPRK1* were closely related to oocyte maturation [32] and maternal behavior [33]. *LPAR3* could interact with multiple reproductive hormones, including progesterone [34], estrogen [35], and prostaglandin [36,37], to affect reproduction. Taken together, our and others’ results suggest that the neuroactive ligand–receptor interaction pathway could play a critical role in affecting goose reproductive performance.

Furthermore, we analyzed intra-breed differences in egg production performance to determine whether there was a difference in levels of mRNAs and lncRNAs involved in the neuroactive ligand–receptor interaction pathway. The number of DEGs and DE lncRNAs identified between different breeds was much higher than that of those between groups showing high- and low egg production performance within the same breed. In particular, only 65 DEGs were identified between high- and low egg production groups within LG. Because the reproductive performance of goose is generally thought to be regulated by the HPG axis [38,39], our results may suggest that the hypothalamus and pituitary should be the key organs controlling egg production performance in LG. Moreover, the expression difference in the DEGs of the neuroactive ligand–receptor interaction pathway was found in the SWG group. In recent years, the neuroactive ligand–receptor interaction pathway was also suggested to be involved in regulating egg production performance in both chickens [7] and ducks [5]. These results suggested that the neuroactive ligand–receptor interaction pathway was more active in individuals with high egg production performance than those with low egg production. Meanwhile, some DE lncRNAs between these two breeds were enriched in several immune-related pathways, including influenza A, Fanconi anemia, herpes simplex infection, RIG-I-like receptor signaling, and Toll-like receptor signaling pathways. The innate and acquired immunity in the poultry ovary is closely related to reproductive performance. For example, the TLR signaling pathway was demonstrated to affect granulosa cell steroidogenesis, differentiation, and apoptosis in response to environmental stimuli [40]. These results indicated that differences in immunity within the same breed may affect egg production performance.

Our results could also explain why there were differences in the time of egg production initiation between SWG and LG. Expression of *HTR1F*, *HTR1B*, and *HTR7*, three members of the HTR family, was significantly different between these two breeds at the age of 145 days. HTR is the receptor for serotonin (5-HT), which exerts its effects by binding to HTR. Brain-derived 5-HT accounts for approximately 5% of the total body serotonin [41]. Previous reports have shown that brain-derived 5-HT inhibits poultry reproductive performance by inhibiting GnRH synthesis or LH secretion [42,43]. The remaining 95% of 5-HT is produced by peripheral organs, and in recent years, 5-HT was also shown to be able to regulate the organism’s metabolism [44]. Compared to LG, expression of *HTR2B* and *HTR7* was upregulated in SWG, while that of *HTR1F* was downregulated in SWG. A previous study has shown that 5-HT stimulates lipolysis in white adipocytes by acting through its receptor *HTR2B* to activate hormone-sensitive lipase [45]. Adipocyte-specific ablation of *HTR2B* resulted in a reduction in FFA and glycerol levels in the blood of fasted mice [46]. Additionally, stimulation of the *HTR2B* receptor by gut-derived 5-HT might also suppress lipogenesis, because another research group [47] showed that ablation of the *HTR2B* signaling during in vitro adipocyte differentiation resulted in triglyceride accumulation. These results suggest that HTR may affect goose egg production performance by regulating stromal metabolic functions. Most of the DEGs between LG and SWG were concentrated in metabolism-related pathways at 145 days. The DEGs enriched in the lipid metabolic pathways were significantly upregulated in LG. Among them, *SCD5* has been reported to promote lipogenesis [48,49]. Upregulated levels of *SCD5* in LG indicate that LG may have a stronger capacity for fat deposition compared to SWG, which was also reflected by different responses of their livers to overfeeding [50]. Previous studies on poultry have shown that excessive fat deposition can delay the onset of egg production [51,52]. Therefore, the excessive fat deposition in LG may be one of the reasons why LG begins to lay eggs earlier than SWG.

In addition, obesity is thought to be one of the factors affecting reproductive function [51,53]. Different expression patterns of *HTR7 and HTR1B* were observed at each studied age between SWG and LG. A previous study has shown that expression of *HTR1B* increased significantly when mice were given a drug to treat obesity [54]. As an obesity-related gene, *LEPR* is also thought to be a mediator between reproduction and metabolism [55], and it was observed to be significantly and differently expressed in the stroma between SWG and LG. Next, we found that lncRNA.MSTRG.1426.1 could target both *HTR1B* and *HTR7*. *HTR1B* was found to be regulated by some DE lncRNAs. Several previous studies have indicated that lncRNAs are conserved in their functions [56]. Functional conservation, despite variations in sequence, is a characteristic of lncRNAs [57]. In addition, the Wnt signaling pathway, mTOR signaling pathway, and MAPK signaling pathway were enriched in LLD vs. LSC and HLD vs. HSC groups. In these pathways, *BAMBI*, *WNT9A*, *SFRP1*, *PRKCB*, *SGK1*, *DUSP8*, and *DUSP6* were upregulated in the SWG group. *BAMBI* [58,59] and *DUSP8* [60] can promote granulosa cell differentiation. These findings suggest that the differentiation ability of granulosa cells in the SWG ovarian stroma is better than that of the LG ovarian stroma at 730 days. Secondly, *WNT9A* [61], *PRKCB* [62], *SGK1* [63], and *DUSP6* [64] have been shown to promote or inhibit the development of ovarian cancer. These results suggest that the number of eggs produced may influence the risk of ovarian cancer [65].

The expression patterns of *HTR1B, HTR1F, HTR2B*, and *HTR7* were not identical. Previous reports indicated that *HTR1B*, *HTR1F*, *HTR2B*, and *HTR7* are GPCRs [66]. Meanwhile, these genes were all predicted to contain a tm_GPCRs superfamily domain in goose. The crystal structures of HTR1B [67] and HTR2B [68] have been parsed, and differences in their binding pockets have been found. However, the physicochemical properties of goose HTR1B and HTR2B proteins are relatively consistent, and similar expression patterns of HTR1B and HTR2B mRNAs were observed in the ovarian stroma between SWG and LG. In contrast to *HTR1B*, *HTR2B*, and *HTR7*, expression of *HTR1F* in the ovarian stroma of SWG is lower than in that of LG at 145 days. By analyzing the secondary structure of the protein, we found that HTR1F had the highest proportion of α helices and the lowest proportion of β turns and random coils among the four genes. The difference in secondary structure may be the reason for the difference in expression.

## 5. Conclusions

In conclusion, both the mRNA and lncRNA expression profiles in the ovarian stroma of Sichuan white goose and Landes goose were examined by RNAseq, along with gene expression profiles at different times within each breed. In silico functional analysis of these DE mRNAs and lncRNAs suggests that the neuroactive ligand–receptor interaction pathway is crucial for egg production, and particularly members of the 5-hydroxytryptamlne receptor (HTR) family affect egg production by regulating ovarian metabolic function. Furthermore, the big differences in the secondary structures of HTR1F and HTR1B, and HTR2B, and HTR7 may account for the different expression patterns in goose ovaries seen in both inter- and intra-breed groups. These results provide novel insights into the mechanisms regulating poultry egg production. We will functionally validate these findings in future studies to gain a deeper understanding of the roles of the HTR family members in poultry egg production.

## Figures and Tables

**Table 1:** Sample grouping information in this study.

Group Name	Control Group	Experimental Group
YLD vs. YSC	LG at 145 days	SWG at 145 days
HLD vs. HSC	LG with high egg production performance at 730 days	SWG with high egg production performance at 730 days
LLD vs. LSC	LG with low egg production performance at 730 days	SWG with low egg production performance at 730 days
LLD vs. HLD	LG with low egg production performance at 730 days	LG with high egg production performance at 730 days
LSC vs. HSC	SWG with low egg production performance at 730 days	SWG with high egg production performance at 730 days

**Table 2:** PCR primers used in this study.

Primer Name	Sequence (5′-3′)	Product Length (bp)
*ACAA1*-F	CGCTTTGGTCGCAAGAGTT	187
*ACAA1*-R	ATTGGCACTTCTGAGGGACAT	
*RPS6*-F	TTGTCCGAATCAGTGGTGGC	121
*RPS6*-R	GTTCTCCTGGGGCGGTAGC	
*GAPDH*-F	CATGTTCGTGATGGGTGTG	239
*GAPDH*-R	CTGGGATAATGTTCTGGGC	
lncRNA.MSTRG.7198.1-F	TCCTTACTCCTGCTTCTACCA	114
lncRNA.MSTRG.7198.1-R	CCTGGCAACTTCTTGTCTGT	
lncRNA.MSTRG.19978.1-F	CCAGACCACAGAGCCAAACA	100
lncRNA.MSTRG.19978.1-R	CCCCCAGACATCAGCAAGAG	
lncRNA.MSTRG.11170.1-F	AGTGAGAGGAGTGAGGAACAG	129
lncRNA.MSTRG.11170.1-R	GGACAGCCTGCTTCACC	
*HTR7*-F	GCAGCCCTCCAACTATCTC	225
*HTR7*-R	AGAGGTCTTGTTATTCCCAGG	
*HTR1F*-F	CTGTAGCCCTGCCTTCTCCC	99
*HTR1F*-R	GTGGCTCGCTATGAACTGGTAAC	
*HTR1B*-F	TTCCCCACTTTGCTGCTGATA	108
*HTR1B*-R	AGCCCGAGTTAGTCTTTTACCC	
*HTR2B*-F	GAACCTCACTCTAAAGGGGAC	187
*HTR2B*-R	ATGGTAAACTGGTCATCTGCTA	
*β-actin*-F	CAACGAGCGGTTCAGGTGT	99
*β-actin*-R	TGGAGTTGAAGGTGGTCTCGT	

## Supplementary Materials

The following are available online at https://www.mdpi.com/2073-4425/11/4/455/s1. Table S1: Basic information on the sequencing data of all samples in this study, Table S2: KEGG pathways enriched by the DEGs in different breeds and related information, Table S3: Significantly differentially expressed mRNAs in the different groups, Table S4: Significantly differentially expressed lncRNAs in the different groups, Table S5: Cis-target mRNAs of the differentially expressed lncRNAs in different groups, Table S6: Trans-target mRNAs of the differentially expressed lncRNAs in different groups, Table S7: GO terms of the mRNAs in group YLD vs. YSC, Table S8: GO terms of the mRNAs in group HLD vs. HSC, Table S9: GO terms of the mRNAs in group LLD vs. LSC, Table S10: GO terms of the mRNAs in group LLD vs. HLD, Table S11: GO terms of the mRNAs in group LSC vs. HSC.
