# Supplementary material for: Comparative Transcriptome Analysis Suggests Key Roles for 5-Hydroxytryptamlne Receptors in Control of Goose Egg Production
Source: Genes (Basel). 2020 Apr 22;11(4):455. doi: 10.3390/genes11040455 (PMC7230923; doi:10.3390/genes11040455)
Supplement: Supplementary file 1 [file genes-11-00455-s001.zip › 4.Supplementary table/Supplementary table 1.docx]

Supplementary table 1. Basic information of sequencing data of all samples in this study

| Sample | OD260/280 | OD260/230 | RIN | Raw Reads | Clean Reads | SRR ID | Q20(%) | Q30(%) | GC Content(%) | mapping（%） |
| --- | --- | --- | --- | --- | --- | --- | --- | --- | --- | --- |
| Lsc_3 | 1.96 | 2.31 | 9.00 | 55,222,827 | 54,440,160 | SRR10867441 | 95.10 | 88.50 | 51.15 | 79.37 |
| Lsc_1 | 1.87 | 2.01 | 8.60 | 58,506,268 | 57,679,319 | SRR10867446 | 95.18 | 88.60 | 50.95 | 78.55 |
| Lsc_2 | 1.89 | 2.20 | 9.10 | 44,531,618 | 43,935,609 | SRR10867444 | 97.34 | 93.33 | 50.36 | 83.16 |
| Hsc_1 | 1.94 | 1.99 | 8.30 | 51,648,658 | 50,735,175 | SRR10867445 | 95.14 | 88.58 | 51.70 | 81.67 |
| Hsc_3 | 1.93 | 2.02 | 9.10 | 55,458,801 | 54,630,175 | SRR10867442 | 95.16 | 88.56 | 51.91 | 81.04 |
| Hsc_2 | 1.93 | 2.37 | 8.60 | 56,273,798 | 55,562,582 | SRR10867443 | 95.09 | 88.47 | 51.82 | 78.24 |
| Lld_2 | 1.83 | 1.49 | 8.30 | 52,976,463 | 50,953,947 | SRR10867436 | 97.06 | 92.69 | 50.66 | 80.34 |
| Lld_3 | 1.96 | 2.06 | 9.00 | 48,453,916 | 47,552,156 | SRR10867434 | 97.10 | 92.75 | 50.52 | 80.46 |
| Lld_1 | 1.92 | 1.24 | 8.60 | 57,606,694 | 56,683,820 | SRR10867439 | 96.10 | 90.60 | 51.39 | 76.31 |
| Hld_3 | 1.92 | 2.08 | 8.40 | 45,856,569 | 45,269,109 | SRR10867435 | 96.05 | 90.50 | 51.94 | 78.51 |
| Hld_1 | 1.80 | 2.08 | 10.00 | 55,236,578 | 54,303,473 | SRR10867438 | 96.06 | 90.50 | 51.75 | 74.43 |
| Hld_2 | 1.96 | 2.14 | 9.70 | 49,612,759 | 49,074,801 | SRR10867437 | 96.05 | 90.44 | 50.02 | 79.62 |
| Ysc_1 | 1.84 | 1.91 | 7.80 | 54,384,162 | 53,738,097 | SRR10867433 | 96.09 | 90.50 | 49.05 | 82.39 |
| Ysc_2 | 1.93 | 2.04 | 9.80 | 54,470,559 | 53,885,827 | SRR10867448 | 96.03 | 90.40 | 50.35 | 82.98 |
| Ysc_3 | 1.92 | 2.08 | 9.10 | 63,883,470 | 62,986,615 | SRR10867447 | 96.00 | 90.33 | 49.96 | 81.91 |
| Yld_1 | 2.05 | 2.08 | 9.70 | 57,724,110 | 56,715,870 | SRR10867450 | 96.91 | 92.41 | 52.58 | 78.17 |
| Yld_2 | 1.99 | 2.07 | 9.00 | 47,187,233 | 46,629,792 | SRR10867449 | 97.06 | 92.75 | 51.88 | 79.79 |
| Yld_3 | 1.88 | 1.91 | 9.20 | 43,777,661 | 43,269,938 | SRR10867440 | 97.00 | 92.59 | 51.36 | 77.31 |
