# Supplementary material for: Comparative Transcriptome Analysis Suggests Key Roles for 5-Hydroxytryptamlne Receptors in Control of Goose Egg Production
Source: Genes (Basel). 2020 Apr 22;11(4):455. doi: 10.3390/genes11040455 (PMC7230923; doi:10.3390/genes11040455)
Supplement: Supplementary file 1 [file genes-11-00455-s001.zip › 4.Supplementary table/Supplementary Table. 2.docx]

Supplementary table 2. KEGG pathway enriched by DEGs in different breeds and related information

| KEGG_Name | Input number | Backeground | *P*_value | DEGs | groups |
| --- | --- | --- | --- | --- | --- |
| Neuroactive ligand-receptor interaction | 8 | 267 | 0.001 | *HTR2B, P2RY13, F2RL2, GLRA2, OPRM1, HTR7, OPRK1, PTGER3* | yld VS ysc |
| Metabolic pathways | 18 | 1092 | 0.001 | *GALNT9, AK5, SMPD3,AGXT2, DHCR24, GAD2, MGLL, FDPS, SGSH, MSMO1, NFS1, HMGCS1, SI, ALDH3B1, ACAA1, MTAP, GAPDH, ST8SIA1* |  |
| Valine, leucine and isoleucine degradation | 3 | 47 | 0.006 | *AGXT2, HMGCS1, ACAA1* |  |
| Steroid biosynthesis | 2 | 17 | 0.008 | *DHCR24, MSMO1* |  |
| PPAR signaling pathway | 3 | 60 | 0.010 | *RXRG,SCD5, ACAA1* |  |
| Terpenoid backbone biosynthesis | 2 | 20 | 0.012 | *HMGCS1, FDPS* |  |
| Biosynthesis of unsaturated fatty acids | 2 | 22 | 0.013 | *SCD5, ACAA1* |  |
| Tight junction | 4 | 122 | 0.013 | *PRKCH, MYH7B, OCLN, YBX3* |  |
| Butanoate metabolism | 2 | 25 | 0.016 | *HMGCS1, GAD2* |  |
| beta-Alanine metabolism | 2 | 27 | 0.018 | *GAD2, ALDH3B1* |  |
| Alanine, aspartate and glutamate metabolism | 2 | 32 | 0.025 | *AGXT2, GAD2* |  |
| Calcium signaling pathway | 4 | 161 | 0.031 | *CACNA1E, HTR2B, HTR7, PTGER3* |  |
| Cysteine and methionine metabolism | 2 | 39 | 0.035 | *AGXT2, MTAP* |  |
| Fatty acid metabolism | 2 | 42 | 0.040 | *SCD5, ACAA1* |  |
| Phenylalanine metabolism  Calcium signaling pathway  Neuroactive ligand-receptor interaction  Tyrosine metabolism  Cysteine and methionine metabolism  Wnt signaling pathway  MAPK signaling pathway  Glycolysis / Gluconeogenesis  Phenylalanine, tyrosine and tryptophan biosynthesis  Glycerolipid metabolism  Endocytosis | 2  4  5  2  2  3  4  2  1  2  4 | 15  161  267  33  39  126  223  51  5  54  238 | 0.003  0.010  0.012  0.013  0.018  0.027  0.027  0.029  0.030  0.032  0.034 | *TAT, ALDH3B1*  *NOS1, HTR7,SLC8A1, PPP3CB*  *GLRA2, GRM4, HTR1B, HTR7, LEPR*  *TAT, ALDH3B1*  *TAT, MTAP*  *WNT9A, SFRP1, PPP3CB*  *DUSP8, DUSP6, PPP3CB, TGFBR1*  *PGM1, ALDH3B1*  *TAT*  *MOGAT1,GLYCTK*  *VPS36, CHMP4C, TGFBR1, PLD1* | hld VS hsc |
| Wnt signaling pathway  Neuroactive ligand-receptor interaction  Ether lipid metabolism  Sphingolipid metabolism  mTOR signaling pathway  PPAR signaling pathway  Adipocytokine signaling pathway  Calcium signaling pathway  Sulfur relay system | 4  5  2  2  3  2  2  3  1 | 126  267  43  47  142  60  65  161  9 | 0.004  0.010  0.020  0.023  0.033  0.036  0.041  0.045  0.047 | *BAMBI, WNT9A, SFRP1, PRKCB*  *HTR1F, LPAR3, HTR1B, AVPR1A, GABRP*  *UGT8, GAL3ST1*  *UGT8, GAL3ST1*  *WNT9A, PRKCB, SGK1*  *SCP2, CD36*  *G6PC2, CD36*  *PRKCB, AVPR1A, RYR3*  *MOCS3* | lld VS lsc |
